# Maternal deaths in eastern Indonesia: 20 years and still walking: an ethnographic study

**DOI:** 10.1186/1471-2393-14-39

**Published:** 2014-01-22

**Authors:** Suzanne Belton, Bronwyn Myers, Frederika Rambu Ngana

**Affiliations:** 1 Menzies School of Health Research, Casuarina, Darwin, NT, Australia; 2 Department of Research Institute for the Environment and Livelihoods, Charles Darwin University, Casuarina, Darwin, NT, Australia; 3 Nusa Cendana University, Kupang, Nusa Tenggara Timur, Indonesia

**Keywords:** Maternal mortality, Indonesia, Stigma, Health-seeking, Fatalism, Geo information systems

## Abstract

**Background:**

The delays in receiving adequate emergency maternal care described by Thaddeus and Maine twenty years ago are still occurring, as exemplified in this study of cases of maternal deaths in a subdistrict in rural eastern Indonesia.

**Methods:**

An ethnographic design was conducted, recruiting eleven families who reported on cases of maternal deaths in one sub-district of Indonesia, as well as assessing the geographical and cultural context of the villages. Traditional birth attendants and village leaders provided information to the research team which was thematically and contextually analysed.

**Results:**

Two stages to the first and second delays have been differentiated in this study. First, delays in the decision to seek care comprised time taken to recognise (if at all) that an emergency situation existed, followed by time taken to reach a decision to request care. The decision to request care resided variously with the family or cadre. Second, delays in reaching care comprised time taken to deliver the request for help and then time for help to arrive. A phone was not available to request care in many cases and so the request was delivered by walking or motorbike. In two cases where the decision to seek care and the delivery of the request happened in a timely way, help was delayed because the midwife and ambulance respectively were unavailable.

**Conclusions:**

This study, although a small sample, confirmed that either a single delay or a sequence of delays can prove fatal. Delays were determined by both social and geographic factors, any of which alone could be limiting. Initiatives to improve maternal health outcomes need to address multiple factors: increased awareness of equitable access to maternal health care, village preparedness for emergency response, improved access to telecommunications and geographic access.

## Background

Maternal mortality remains a tenacious problem in Indonesia despite concerted efforts by government and non-government sectors. Maternal mortality ratio (MMR) in Indonesia decreased and then plateaued in the past 20 years, with the number of maternal deaths per 100,000 live births decreasing from 390 in 1991, to 228 in 2007, and 220 in 2010, twice the Millennium Development Goal for 2015 of 102 [1,2]. The eastern Indonesia province of Nusa Tenggara Timur (NTT) still experiences high levels of maternal mortality. The MMR for NTT province remains higher than the national average, with 271/100,000 live births in NTT in 2010 [3], and the rural district of South Central Timor (TTS) is particularly high with MMR of 596 in 2010 [3].

The challenges of delivering adequate maternal health care in Indonesia are well known and include limited access to quality facilities, limited availability of health staff, lack of awareness and cultural constraints regarding safe motherhood, low nutritional and health status of women, unmet need for contraception, and a frail system for recording maternal deaths [1]. Skilled attendance at delivery is recognised as the most important single factor for reducing maternal mortality [4]. Official data for 2010 indicate, in Indonesia and NTT province respectively, 82% and 76% of births were assisted by a skilled birth attendant [5]. Studies in Indonesia have shown that women who receive antenatal care are more likely to have skilled attendance at birth [6]. Antenatal care is generally infrequent for women from rural areas, from poor households and with little education [7] and women have particular preferences for birth at home [8].

Thaddeus and Maine’s paper ‘Too Far to Walk’ in 1994 evocatively described the multiple factors which can act as barriers to accessing emergency maternal care, identifying three types of delays, in (i) the decision to seek care, (ii) arrival at a health facility; and (iii) the provision of adequate care [9]. Over a decade later in this journal, Gabrysch & Campbell in 2009 identified 20 determinants of use of maternal health services and categorised them into four themes: social-cultural factors; perceived benefit/need; economic accessibility and physical accessibility. They observed that the delay in the decision to seek care is determined by socio-cultural factors, and the delay in reaching a health facility is strongly influenced by economic and geographic factors [10].

Our study focuses on a sub-district in NTT province where terrain is rugged and roads are poor and so geographic barriers are likely to be a barrier to accessing emergency health care (second delay). However some maternal deaths occur relatively close to health facilities suggesting that delays in seeking emergency care (first delays) were significant. The aim of this study was to describe limitations or barriers to accessing emergency maternal care for cases of maternal death in a rural sub-district in NTT. We wished to describe the circumstances of maternal deaths through ethnographic methodology [11] in which the families explained health-seeking behaviours and their perceptions of the causes of birth complications, as well as their access to health care during pregnancy and the maternal emergency. We explored the reasons for choosing the birth place and selecting a birth attendant, as well as decisions about birth preparations and access to Indonesian social welfare in the form of the health care card (*Jamkesmas*, JPS). Our frame of study was within the extended family unit and set at the village level.

## Methods

### Study site and sampling methods

This study was conducted in Nusa Tenggara Timur (NTT) province, eastern Indonesia, one of the poorest provinces in Indonesia. In 2010, NTT had a population of 4,619,655 spread through 20 districts (*kabupaten*) and 1 city, 287 sub-district (*kecamatan*) and 2,539 villages [12]. At this time, in this sub-district 4 of the 11 villages had resident midwives (see Table 1 Subdistrict B, in Rambu Ngana et al. 2012, page 812 [13]). The majority of the people are Christian and speak Bahasa Indonesia, however there is a diversity of languages, religions and ethnic groups. Agriculture is the occupation of the majority of the population, and light industry and mining are minor sources of livelihoods.

**Table 1:** Interview topics covered in interviews with family members and village leaders

**Participants**	**Interview topics**
Family Members	Social demographics; reproductive history; condition of infant; process of death; perceptions on the cause of death; access to health services, choice of birth place and assistants; preparation for birth; use of emergency services; responsiveness of the health system.
Village Leaders	Condition of roads; seasonal access by road; access to clean water; availability of electricity; nutritional security; access to school; mobile phone coverage or public phone; ethnic diversity and religion, languages; ‘normal’ birth practices; availability of health insurance; access to health facilities; presence of a midwife; access to ambulance service.

One sub-district was purposively sampled as it had a high maternal mortality ratio and the authors had established collaborative working relations with leaders in health and social welfare in this sub-district. The study sub-district is located in a district with a total population of 435,000, a population of fertile age women of about 100,000, and a population density of 110 per km^2 ^[12]. This sub-district is characterised by mountainous terrain, distinctive one wet and dry seasons each year, and roads that are often cut by flooding rivers and landslides in the wet season.

### Participants

A female church leader invited the research team to work in her area. In the month prior to the research team arriving, she sent messages to eleven families who had experienced a maternal death inviting them to join the study and no family refused. Access to many of the villages was difficult. Road conditions were very poor and in some cases there were no roads. The research team drove in a car where possible and walked along river beds and into the hills to speak with respondents.

The participants included the near relatives of the dead woman and this was most commonly her husband, mother, mother-in-law, sisters and brothers and relatives of her husband. Often the family members interviewed included traditional midwives (*dukun*). The research team wished to speak with the people who had made decisions regarding accessing maternal health care and who had provided direct care during the birth and the postnatal period. No doctors or midwives were interviewed. Village leaders and their deputies were also interviewed about the organisational structure and infrastructure of the village and the process of reporting deaths in the village.

### Data collection and analysis

Our Indonesian academic colleagues invited us to the location. The research team included one educated older man who spoke the local language, one female leader who spoke the local language, one female academically trained researcher who spoke the local language, one academically trained researcher who spoke Bahasa Indonesia and English, and two Australian academics trained respectively in medical anthropology and geography. We trained together prior to data collection in role plays, practice interviews and discussions on maternal mortality and research ethics. After training and sharing cultural information, the research team travelled to villages and were invited into the family homes.

Ethical clearance was obtained from the Human Research Ethics Committee (#H10081) Charles Darwin University and permission was gained from the Indonesian university involved in this research. While Human Research Ethics Committees require written plain language statements and contractual consent forms, the research team found these inappropriate to use in practice. The majority of the respondents were illiterate and preferred their traditional custom for talking with strangers. This involved the visitors (the research team) greeting all family members and providing an explanation of the visitors’ intentions, usually in the local language. The visitors offered betel nuts and lime powder (*sirih pinang*) on woven boxes to all members of the family. Acceptance of the betel nut was an indication that consent for the interview had been given [14].

The families preferred to speak as a group and not individually, and groups interviewed ranged from two to 12 people. As questions were answered the family discussed and came to a consensus answer. Often the interviewers encouraged women to speak, as men tended to dominate the conversation.

Key locations in the stories of maternal deaths were recorded with a geo-positioning (GPS) device, including the hospital, health centres (*puskesmas*), health posts (*posyandu*), public phones, and homes where maternal deaths had occurred.

Official data indicated that 11 maternal deaths had occurred, across 11 villages, in the study sub-district during the period 2008 to 2010. We interviewed eight families who had experienced a maternal death, and the village leaders (*kepala desa*) of the five villages where these families were located. The interviews were conducted with all the research team present in June 2011 with families who had experienced maternal death nine months to three years before.

We used a modified interview schedule that had been field-tested in South Sulawesi [6]. Each interview took one to two hours. The interviews with family members and village leaders covered the topics outlined in Table 2. The families’ responses were recorded in the local language and translated into English and coded. A rapid ethnographic method was used and a thematic analysis was undertaken. Our detailed methodology is published in a book titled *Discourse, power, resistance – Down Under*; a collection of papers presented at the Association for Qualitative Research conference in 2012 [15].

**Table 2:** Information about cases of maternal death and the delays in getting emergency health care

**Case**	**First delay**		**Second delay**		**Third delay – adequate care**	**Time from delivery to death**	**Age of woman**	**Prior live births**	**High risk?**	**Antenatal care?**	**Married?**	**Baby survived?**
	**→in decision to seek care**		**– in reaching care**									
	**Emergency recognised?**	**Was help sought?**	**Delays in transmitting message seeking help?**	**Delays in help arriving?**	**Was care effective?**							
1	**Delayed**	Yes	**Yes, walked to traditional birth attendant**	**Yes, walked**	No care received	3 hour	20	0	No	No	No	Yes
3	**Delayed**	Yes	**Yes, walked**	**Yes, walked**	No care received	4 days	27	0	No	Yes	Yes	Yes
4	**Delayed**	**No, wet season travel too difficult**	No care sought	No care received	5 days	29	0	No	Yes	Yes	2 months	
7	Yes	**No, ashamed**	No care sought	No care received	1 day	28	0	No	No	No	Yes	
8	Yes	**Yes, delayed, cadre said wait until dawn**	No, midwife phoned (at dawn)	No, midwife came immediately by motorbike, arrived after death	No care received		24	1	No	Yes	Yes ^	Yes
6	Yes	Yes	**Yes, walked to midwife**	**Yes, midwife at another birth**	No care received	1 hour		3	No	Yes	Yes #	Yes
5	Yes*	Yes	No, phoned clinic	**Yes, ambulance broke down**	No care received	2 days		4	Yes, twins	Yes	Yes	Survived birth, one died later
2	Yes	Yes	**Some delay, motorbike to clinic**	No	Hospital care not successful	2 days	27	3	**Yes, prior illness**	Yes	Yes	1 hour

*Nurse present, no training birth assistant at all other births.

#Traditional marriage ceremonies not completed.

## Results

### Information from village leaders or their representatives

The six villages had populations ranging from 1700–2300, comprising 400–700 households. Subsistence farming was most common (~95%) with other occupations including teachers, public servants, and motor bike taxi drivers. Staple foods were maize and cassava, and the busiest periods for farming activities were August to October for preparing fields, and April for harvesting maize. The most common language spoken was Dawan and some villagers did not speak Bahasa Indonesia. The villages each had between one and three primary schools, and one junior high school. Malnutrition was identified as a common problem and a high proportion of the villagers were classified as living in poverty. All villages had one public phone at a central location however in many cases this phone was not working. Mobile phone signal varied amongst locations and was generally strong enough for text messages, although only on hilltops in some villages. One village had no electricity; no functioning solar panels and was unable to afford to run generators. In this village there was no power for recharging mobile phones although the signal was adequate.

All villages experienced restriction of access by road due to seasonal flooding, landslides and damage to roads. The heaviest and most persistent rains were typically experienced from January to April, with consequent closure of roads often experienced in April and May. The interviews were conducted in June 2011, after an exceptionally wet period, and many roads were damaged, and some remained cut by landslides.

The villages were all between 10 and 30 km from the subdistrict capital (where a health clinic is located), however rugged terrain and poor roads meant that average speed by car was less than 10 km/hr, so that travel time by car from these villages to the subdistrict clinic was between one and four hours. Some villages had a bus service running each day and trucks travelled from the village to a local market in a nearby village once a week. All villages had some motorbike taxis but one of the villages had just one motorbike. No villages permanently housed cars. Ambulances from the subdistrict clinic were called in some medical emergencies however all motorised transport was limited by the state of the roads, and only as far as where roads were cut by floodwaters at times. Villagers commonly walked to visit neighbouring villages and markets. In medical emergencies when roads were cut, patients were carried by a group of family members, either in a sling or chair supported by poles.

Most villages comprised three or four hamlets (*dusun*), with most but not all hamlets having a health post (*posyandu*). If there was no resident midwife in the village, a midwife from the subdistrict clinic visited each *posyandu* in the village on one day each month. In most villages, most of the households had a health care card (*Jamkesmas*, JPS) and, in one village where few people had a JPS card, the village leader believed most households were eligible to have one. Holders of JPS were entitled to free health care at the subdistrict clinic, and those without a card were required to pay Rp1,000 (US 10 cents) per visit. JPS holders were also entitled to free ambulance services if needed, however users commonly felt obliged to pay for fuel or to pay the driver (approximately USD $10-30) if they used these services. One village leader said that some villagers might visit the *posyandu* but were “ashamed” to visit the subdistrict clinic because of lack of privacy there.

A system of saving for expenses associated with birth (*tabulin*) had been set up in many villages. Under this system, when a woman becomes pregnant she can put aside money with the cadre each month during the pregnancy. The amount varied amongst villages from IDR 2,000 to 20,000 (USD 0.2-2) per month. In half the villages investigated, the system had been introduced recently and was not widely supported partly due to distrust. In a village where a midwife was resident, the midwife made the preparations for a potential maternal emergency during the first antenatal visit by collecting the names and phone numbers of about 20 male relatives of the woman, including her husband, so that she could call on them to carry the woman to where an ambulance could meet them if needed. None of the villages appeared to have an active system of village preparedness (*desa siaga*).

When asked about the usual actions at a birth, the village leaders mentioned the officially supported actions of moving to the clinic near the expected time of the birth, and seeking the assistance of the midwife. This is a requirement under the health department’s program mother and child health (*Revolusi KIA*) which has been implemented since 2009. However, many village leaders also mentioned that most women give birth in the traditional house (*rumah bulat*) and are often assisted by a traditional birth assistant (*dukun)*. All village leaders confirmed that since 2010 there is a fine for giving birth at home of IDR 250,000 or 500,000 (USD 25 or 50) and almost all admitted that the fine was rarely enforced.

If a maternal death occurs, usually the midwife will report this death to the subdistrict clinic, and the cadre or Head of the hamlet (*Bapak dusun*) reports to the village leader, who in turn reports the death to the Subdistrict Leader (*Camat*). Villages also have a system of announcing the death by ringing of bells or sounding of notes on buffalo horns, plus announcements at church services.

### Information from families

Eight families were contacted. Due to the terrain and logistics we were unable to contact all families in our sample. As families recounted the events that surrounded the women’s deaths several themes emerged: remoteness; poverty; traditional customs; health system dysfunction and fatalism.

### Remoteness

While the houses where maternal deaths occurred were between 5 and 15 km from a health clinic and about 35 km from emergency maternal care, these locations can be considered remote from care because travel was slow and telecommunication was limited. In many cases a mobile phone was not available or there was no mobile phone signal so that family members walked to the traditional birth attendant or village midwife to seek care.

Husbands described rivers that rose and would endanger lives if an attempt was made to cross.

Interviewer: What I mean is why didn’t you take her to the village health post in [name of village]? Why didn’t you take her to [location of health post] to give birth?

Husband: It was raining ma’am, how could we go out? We couldn’t go out of the house, what could we do, we just couldn’t. [Case 4]

During the rainy season in this region, flooding rivers and landslides create barriers to access. We estimated travel time from homes to the closest clinic offering emergency obstetric care and calculated the proportion of the population with more than two hours travel time as a measure of remoteness from EmOC. During the wet season the proportion of the population remote from EmOC by this measure was greater than in the dry season: two times greater for the study sub-district and much greater increases for other sub-districts [*Pending publication*].

### Poverty

All of the families were poor, living in simple housing, many with dirt floors, no electricity, and no sanitation or running water. All were eligible for the *Jamkesmas* card, however only two families held the card which entitled them to free health care, and even they paid IDR 150,000 to 300,000 (USD $16 to 33) to use health care services.

Interviewer: Did you have a *Jamkesmas* card?

Husband: We did not have *Jamkesmas*. They (clinic staff) did not give us a *Jamkesmas* card.

Interviewer: Why didn’t they give you a *Jamkesmas* card?

Husband: We applied for the *Jamkesmas* but the clinic staff hadn’t given it to us. [Case 4]

We noted that bureaucratic delay and village level uncertainty inhibited eligible villagers from holding a health care card.

### Ritual customs of tradition

Women and their families preferred to give birth with their families close in the round houses, the place that houses heirlooms, corn harvest and symbols of fertility, and is the domain of women (as observed by Kambaru Windi & Whittaker [16]. Traditional midwives assisted with cutting the cord and handling the placenta. Members of families were often present during the labour and assisted the women in the post-partum time and prepared special food for the woman. If it was perceived that the women were ill a *naketi* ceremony was performed to promote health and this could delay formal health care seeking. Only one woman in our study had complained of sickness (chest pains) during her antenatal period and she was taken to formal health care by ambulance.

Father in law: She was bleeding more than one hour. Her placenta did not come out. This was the first time this happened to her. She gave birth to three daughters before this baby and she was fine. She said that if she ever gave birth to a baby boy then she will die.

Mother in law: She also said that if she gave birth to a baby girl then she will not die.

Interviewer: Did she know that she would give birth to a baby boy?

Mother in law: Yes, she did.

Father in law: The *dukun* asked her to do *naketi* because the placenta did not come out.

Mother in law: Her mother came and asked us to do *naketi*. Then we did *naketi* ceremony by collecting woven cloth (*tenun ikat)*, plants and chicken but later the family took those back again. Maybe if we finished doing *naketi* ceremony, the midwife could find her still alive. [Case 6]

While ceremonies do no direct harm, they can take up precious time and resources that could be better spent seeking emergency obstetric care.

Two of the women who died were not married, and their families reported that these women were deeply ashamed at being pregnant without having a husband to take responsibility for them. According to relatives, these women declined to see midwives or acknowledge their pregnancies publically. The families reported that the women took over 24 hours to die after they gave birth, probably due to blood loss, and the families were reluctant to pay any fees or seek health care. These deaths had not been reported and were not included in official records. Both families suggested it was the woman’s wish not to receive health care, however dead women cannot tell their own stories.

One brother recounted his dead sister’s words:

Adopted brother: She left if all up to God, after she gave birth, to let her die.

Interviewer: Why did she say that?

Adopted brother: She helplessly (surrendered) to God to let her die when she gave birth. She thought that because she does not have a husband, she does not want to make problems for her family especially for her adopted parents. [Case 7]

In several cases the woman was married according to the law but had not completed the *belis* (bride price) ceremony.

### Health system dysfunction

There were several examples of where the health system had been dysfunctional for these women. In these cases the women were healthy and had regular antenatal checks. For one woman twins were not identified in the antenatal period and only discovered by the family during the birth.

The following excerpt illustrates a sequence of unfortunate events that resulted in a fatal delay in accessing emergency maternal care: torrential downpours, undiagnosed twins, poor haemorrhage management, no telecommunications and a lack of transport.

Husband: When my wife died, there was a doctor and a nurse here. The nurse called three times to the health clinic to send the ambulance to pick up my wife but the car was broken. At the same time, there was a woman who was giving birth in a village not far from us. The ambulance was busy picking up that woman to bring to the clinic but after arriving at the clinic, the car broke down. We were waiting for the car for two days. My wife lost too much blood.

She went to the clinic routinely. We have five children. She always went to the clinic since our first child. Our first child has graduated from the junior high school and the second child has graduated from the primary school. My wife never had problem with her pregnancy before but on this last pregnancy, she said she did not have the same strength to give birth as before. So, I told to her to give birth at hospital (clinic) because we would not be able to get help here. My wife agreed to go to hospital (clinic) so we had already prepared everything such us *bose* (traditional food from Timor island), *periuk* (traditional cooking pot), and *kain* (blanket) to take with us. After she died, we kept those things. Our problem here is the road so it is too far [from the house] and it is difficult to reach the clinic. At the time of the birth, all our family and I were here to take care my wife. My mother helped my wife to give birth. I just hugged my wife. She was bleeding too much like water falls out of a bucket. [Case 5]

The husband went on to recount how they tried to call an ambulance but the telephone reception was poor and it was raining heavily. The family managed to speak with a doctor who suggested raising her legs but according to the family administered no medicine and did nothing more. This was her fifth and last pregnancy to undiagnosed twin baby boys who survived the birth.

In another case, a young woman died of a haemorrhage after retaining her placenta. Despite the family, traditional midwife and a cadre staff being aware that something was amiss, she bled to death.

Mother: Her house is in the valley of the mountain, so she came here two months before she gave birth because she wanted to give birth near the health clinic. When she felt pain in her womb, I called cadre at 11 pm. Cadre sat with us until almost 2 am. We asked the cadre to call the midwife but he said *“It is 1.30 am, it is too late. If we call the respected midwife at this time, she will not answer our call. It doesn’t matter, the sun will rise soon”*. So he made us not call the midwife. I feel foolish now. And then later I said to him *“Please, call the midwife for me”.* But the cadre said *“Hold on, the sun will rise soon, morning is almost here”*. There was much bleeding. There was blood like when we slew an animal, maybe caused by a big injury. Blood was flowing while she was sitting. We used a sack to retain it. Later at 3.30 am, I tried to midwife’s mobile phone number by myself, but at 4 am she died. She (the woman) wanted to call the midwife by herself but at 4 am she looked at the cadre and said “this is a pity” and then she died. [Case 8]

### Fatalism

Some of the families we interviewed suggested it was God’s way and that no-one was at fault, they felt that they had little control over whether their wives survived pregnancy or babies survived birth.

Interviewer: So, what did the clinic staff say?

Husband: They did not say anything. When things like this happen we always think; well God has called back my wife. [Case 4]

In one case the woman who died was not married and her daughter survived the birth. Her mother too had not been married and had died giving birth to her. The family believed the surviving child was cursed with a similar fate.

Adopted brother: Excuse me, the dead woman’s mother also died one month after giving birth. She also did not have a husband. We fostered her daughter (the dead woman). But she (the dead woman) also died after giving birth, and then we took care of her daughter (the baby) now. The baby does not have a father, just like her mother.

Interviewer: So, her story was same as her mother’s story. [Case 5]

In all cases there were delays in accessing emergency maternal care (Table 2). In three cases the need for emergency care was recognised after some time, and this delay was compounded because the request for care could only be delivered by walking to the midwife or traditional birth assistant. For the five cases where the need for emergency care was recognised quickly, there were a range of delays in either or both delivering the request for care and in accessing care. Two families did not seek care because of the stigma of an unmarried pregnancy. One family had to walk to contact the midwife and another contacted the clinic by motorbike. In two cases care was requested by phone but in one of these cases the midwife was at another birth and in the other case the ambulance broke down.

## Discussion

In speaking with these eight families, we aimed to describe limitations or barriers to accessing emergency maternal care for cases of maternal death in a rural and remote area while considering the Three Delays model. D’Ambruoso et al. [17] work on access to maternal health services in Java resonates strongly with this study. These authors take a health planning stance across the health system where as our focus is on village level interventions. We also wished to explore why the location of birth was chosen and the selection of birth attendants. From the composite narratives of the families, many of whom were the birth attendants, various truths emerged which were thematically analysed into themes: remoteness; poverty; traditional customs; health system dysfunction and fatalism.

**Figure 1:**
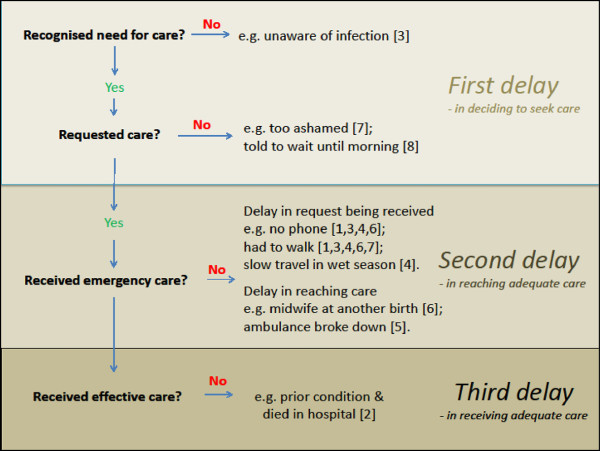
**Cascade of decisions and delays leading to death for the cases in this study (number in square brackets is the case number in Table**1**).**

In almost all cases there was a combination of delays that resulted in a fatal delay in emergency maternal care being received (see Figure 1). Delays in seeking care (first delay) fell into two types: delays in recognising the need for emergency care (e.g. severity of bleeding or infection not recognised); and delays in deciding to seek care (e.g. stigma associated with unmarried pregnancy). Delays in receiving care after the decision to seek care was reached fell into two stages: delays in delivering the request for care (e.g. no phone and walking to the midwife); and delays in help arriving (e.g. the midwife or ambulance being unavailable). Delays at each stage added to the total delay to make it fatal. So, improving access to emergency maternal care requires a combination of approaches which include social and geographic considerations. Addressing just one aspect in isolation will not solve the problem and complex analytical frameworks are required, one example being Price and Hawkins [18].

Although the families acknowledged a preference for traditional home births, most women and their families supported attending antenatal care and were prepared to seek assistance from the midwife and/or clinic once the situation was recognised as an emergency. Families were entitled to social welfare assistance in the form of the health care card but often did not have one. Titaley et al. (*ibid*) also note that villagers in her study were confused about the entitlements provided by a *Jamkesmas* card.

The social stigma and perceived loss of honour an unmarried woman brings to the family caused two women’s deaths. Stigma is defined as ‘an attribute that is deeply discrediting’ [3,19] and in these cases is gendered and culturally situated. Both women were described as attempting to hide their pregnancy and not wanting to draw attention to their pregnant condition by going to the midwife for antenatal care. How true this was cannot be assessed from the families’ accounts. Each family recounted a convoluted story about ineffectual health care seeking at the time of birth, despite traditional and formal midwives being close to hand. Both women took a whole day to die. In one case the village leader called on the house as he was aware that something was amiss, and even he was told that no help was required. These were the most distressing cases to listen to for the research team as these deaths appeared to be avoidable.

The social stigma of unmarried pregnancy is a belief held by many people and Link and Phelan [20] outline various forms of discrimination and the impact they have in public health. And Price (*ibid*) talks of social exclusion that leaves out particular groups from resources and services. These views can be entrenched and stem from patriarchal views about women’s sexuality and roles in society, however it is possible to change and modify these ideas. Village leaders, midwives and doctors may also hold these types of beliefs, that women who conceive out of wedlock are somehow not as precious as other women. These vulnerable women seem to have no one to speak for them. Perhaps this is the first place to start, with education that sensitises leaders, midwives and doctors to seek unmarried women and to place them for special attention due the women’s high risk of neglect by their families. In small interconnected rural villages it is probably not possible to miss gossip about particular women who may be at risk by concealing their pregnancies.

There were several examples of health system dysfunction. Mistakes can happen in any system and it is not surprising that the research team heard cases where this had occurred as we were dealing with cases of maternal death. The most obvious mistake was the case of the undiagnosed twins in a family that would very likely have been willing to take the woman to hospital prior to the late stages of her pregnancy, had they been forewarned. The story of this woman’s management showed further mistakes with mishandling of her haemorrhage, and ineffective emergency transportation. Even though she managed to survive two days after the birth, this was not enough.

While this study only had the accounts of lay people and some official death notification made by staff who were often not present during the death, two causes of death were apparent; haemorrhage and infection. Globally, haemorrhage is the leading cause of death in pregnant women and all staff need to be drilled in the management of antenatal, intrapartum and postpartum haemorrhage. Insertion of IV lines and fluid, as well as oral misoprostol which can be administered in settings outside of hospital by staff who are not necessarily doctors. These types of management need to be commenced prior to emergency evacuations which take many hours in this terrain. Misoprostol can be used to prevent deaths from haemorrhage and there is evidence available regarding its safety and cost effectiveness [21-23].

Another likely cause of death of several women in this study was puerperal infection after a dirty homebirth. In these cases, families who even if they had understood the idea of germs, would not have been able to do a hygienic birth due to the lack of sanitation and running water in the homes. It is not clear from this study why midwives or cadre who live in the villages did not visit women after the homebirth and assess the women’s postpartum recovery. In this area, offering women antibiotics after all homebirths could prevent deaths due to sepsis. Antibiotics prevent infections fulminating to a deadly sepsis. Midwives could give oral or intravenous antibiotics after homebirths [24-26]. Health promotion regarding soap and water washing of hands before helping birthing women may also help.

A sense of fatalism can demotivate individuals who perceive that they have little control over their futures. In Thaddeus and Maine’s 1994 article they postulate that education and modern forms of knowledge may mitigate against fatalistic thinking. As the poorest people on the fringes of Indonesia, who form a minority religious group, it is understandable they feel this way and similarly D’Ambruoso (*ibid*) has recorded fatalistic attitudes in Java. However, mobilisation of villages is possible and people can control some elements of their circumstances. This in part depends on the determination and leadership of these communities. While fatalism is a normal way to relieve grief and guilt about what could have been different, it is unhelpful when considering motivating a village to prepare and act to prevent maternal and infant deaths. Village leaders and midwives need to have the skills to work with communities to address problems that are likely to affect many families. In this area, people are Christian and religious leaders could motivate villagers and discuss God’s will in ways that show that taking initiative is also God’s resolve. Village mobilisation schemes around transportation, saving money and assisting women to go to health services can save lives. The limitations of this study were the small number of families, the focus on village level factors and cross-cultural research within Indonesia; however this study may be similar to other comparable contexts.

## Conclusions

In this investigation of a small sample of maternal deaths in rural villages in eastern Indonesia, there were examples of delays in recognising, seeking and receiving basic emergency obstetric care. Geographic barriers are important and the issue of remoteness is further analysed in another paper [15]. Most women had attended antenatal care and so were engaged with the health care system. However, in all but one case, the realisation that emergency care was needed was delayed: a delay that may have been avoided had a skilled birth attendant been present. The unmarried women were disengaged from the health care system, as they had not attended antenatal care and no midwife was called. Thaddeus and Maine’s model is still relevant today and this study illustrated that multiple social and geographical factors interacted to influence the outcome of the births, including seasonal barriers to transport, entrenched social mores which acted against the best interests of some pregnant women, and a sense of powerlessness to be able to change one’s destiny.

## Competing interests

The research was funded by Charles Darwin University and Menzies School of Health Research. The article processing charge can be paid for by the Menzies School of Health Research. Please note that Suzanne Belton is an Associate Reviewer for BMC Women’s Health. In the past five years the authors have not received any reimbursement, fees, funding or salary from an organisation that may gain or lose from this publication. We hold no stocks or shares that will be affected by this publication. We have no patents relating to this manuscript and we have no competing financial or non-financial interests in the publication of this paper.

## Authors’ contributions

SB and BM have collaborated closely in designing and completing this study. Both are senior researchers in their fields. They shared fieldwork planning, ethic submission writing, data collection and analysis. SB wrote the first draft of this paper and contributed maternal health and anthropological knowledge. BM edited the subsequent draft and contributed human geographical knowledge. FRN interviewed families, translated and provided local information. All authors read and approved the final manuscript.

## Authors’ information

SB is a medical anthropologist with a research interest in sexual and reproductive health. Dr Belton is also a midwife with clinical experience in community health, women’s health, family planning, refugee health and alternative birth systems. Dr Belton’s research interests include the sociology and anthropology of health, the social and cultural context of sexual and reproductive health, gender and violence against women. She is also a specialist in qualitative research methods and has worked in China, Thailand, Indonesia, East Timor and remote Australia.

BM’s early research has been the eco-physiological responses of plants to drought and salinity stress. In the past 15 years, she has gained extensive experience in research and programs for improving livelihoods in eastern Indonesia, including research on fire and resource management and on health mapping. This research has an increasing social science component, and a strong capacity building component, aiming to enable better management of resources at the district level to improve the livelihoods of the rural poor.

FRN is a lecturer at Nusa Cendana University and is currently undertaking a PhD at Charles Darwin University in agent-based modeling to support health service provision in eastern Indonesia.

## Pre-publication history

The pre-publication history for this paper can be accessed here:

http://www.biomedcentral.com/1471-2393/14/39/prepub
